# Middle temporal vein thrombophlebitis, multicompartment deep neck infection, and Lemierre syndrome following temporal lifting: a previously unreported complication chain

**DOI:** 10.1093/bjrcr/uaag010

**Published:** 2026-04-02

**Authors:** Adil Aytaç, Bahar Yanık Keyik

**Affiliations:** Department of Radiology, Faculty of Medicine, Balikesir University Health Practice and Research Hospital, 10145 Balikesir, Turkey; Department of Radiology, Faculty of Medicine, Balikesir University Health Practice and Research Hospital, 10145 Balikesir, Turkey

**Keywords:** temporal lifting, deep neck infection, Lemierre syndrome, temporal vein thrombophlebitis

## Abstract

Temporal lifting is widely performed as an aesthetic procedure with a generally favorable safety profile, yet rare and clinically significant complications may arise due to the anatomical continuity between the temporal region and deep cervical spaces. This report presents an uncommon case in which a healthy woman developed a rapidly progressive sequence of middle temporal vein thrombophlebitis, multicompartment deep neck infection, and Lemierre syndrome (an infectious thrombophlebitis of the internal jugular vein often leading to systemic septic embolization) shortly after temporal lifting. Her symptoms evolved from localized temporal discomfort to trismus, swallowing difficulty, and extensive cervical inflammatory spread, prompting suspicion for deep neck involvement. Magnetic resonance imaging provided critical diagnostic clarity by demonstrating venous thrombosis, diffuse soft tissue infiltration, and narrowing of the parapharyngeal airway. Early administration of broad spectrum antimicrobial therapy, therapeutic anticoagulation, and close respiratory monitoring resulted in full clinical recovery. This case emphasizes that even superficially performed facial rejuvenation procedures may trigger extensive infectious and vascular processes through established fascial pathways. To the best of current knowledge, this is the first reported occurrence of the combined presentation of temporal venous thrombophlebitis, widespread deep neck infection, and Lemierre syndrome following temporal lifting. These observations highlight the importance of early imaging and clinical vigilance in patients presenting with disproportionate postoperative symptoms.

## Introduction

Temporal lifting is an aesthetic surgical procedure that elevates the lateral eyebrows and repositions the overlying temporal skin and soft tissues, producing a more youthful, dynamic, and rested appearance of the upper face. With technological advances in aesthetic surgery and the growing demand for facial rejuvenation, the frequency of this procedure has increased considerably in recent years.^1^ Although the overall incidence of complications is low, temporal lifting may result in bleeding, infection, transient sensory disturbance, swelling, ecchymosis, asymmetry, or scar formation.^1^^,^^2^

Complications associated with temporal lifting and brow lift techniques involving the temporal region are generally reported at low rates within large case series or review articles, whereas individual case reports remain extremely scarce.^1^^,^^3^ The literature includes only isolated descriptions of superficial temporal artery aneurysm and superficial temporal artery arteriovenous fistula related to this procedure.^4^^,^^5^ Additionally, some clinical series have noted that complications such as asymmetry and alopecia may occur at rates ranging between 0.7% and 8.5%.^3^

A comprehensive review of the literature revealed no previously documented case in which temporal lifting was followed by middle temporal venous thrombophlebitis, multicompartment deep neck infection, and subsequent Lemierre syndrome (an infectious thrombophlebitis of the internal jugular vein often leading to systemic septic embolization). In this report, we describe an exceptional clinical course that began with middle temporal vein thrombophlebitis after the procedure and progressed to cellulitis, myositis, widespread deep neck infection, and ultimately Lemierre syndrome, a sequence not previously recorded. This case highlights the decisive role of diagnostic imaging in early detection, confirmation of disease extension, and characterisation of the evolving complication chain, while also drawing attention to rare yet potentially severe infectious and vascular processes that may emerge following this increasingly common aesthetic intervention.

## Case presentation

A 41-year-old woman presented to the otolaryngology clinic with swelling and pain around the right temporal region and auricular area, accompanied by difficulty swallowing. These symptoms were associated with trismus, right submandibular swelling, and tenderness. She denied fever, weight loss, fatigue, or other systemic complaints. She had no history of smoking or alcohol use, no known chronic medical condition, and no regular medication use. The patient had undergone a temporal lifting procedure four days earlier.

Physical examination by the otolaryngologist revealed normal findings in the oral cavity as well as the external, middle, and inner ear. Neurological examination was unremarkable. Because of the presence of trismus and clinical concern for deep neck infection, contrast-enhanced magnetic resonance imaging of the neck was performed.

**Figure 1.**
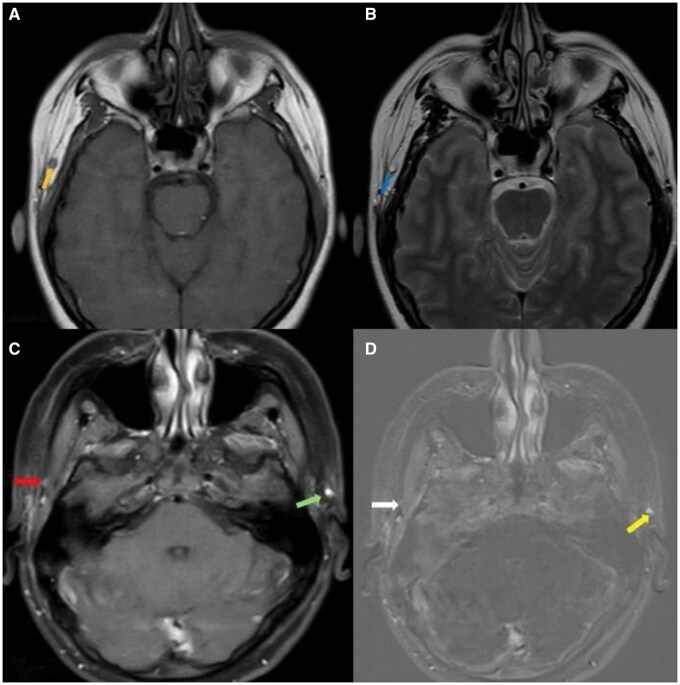
Axial non–fat-suppressed T1-weighted (A) and T2-weighted (B) MR images demonstrating thrombophlebitis of the middle temporal vein, with post-contrast and subtraction correlation (C, D). (A) On the axial T1-weighted image, the yellow arrow indicates loss of normal flow-related signal void within the middle temporal vein. (B) On the axial T2-weighted image, the blue arrow similarly highlights the absence of the expected signal void in the same venous segment, consistent with acute thrombotic occlusion. (C) On the axial post-contrast T1-weighted image, the red arrow indicates absence of luminal contrast enhancement within the right temporal vein, consistent with a filling defect, while the light green arrow demonstrates normal contrast opacification of the contralateral temporal vein for comparison. (D) On the axial subtraction image, the white arrow highlights absence of luminal contrast enhancement within the affected right temporal vein, whereas the light yellow arrow indicates preserved normal luminal contrast enhancement in the contralateral vein.

**Figure 2.**
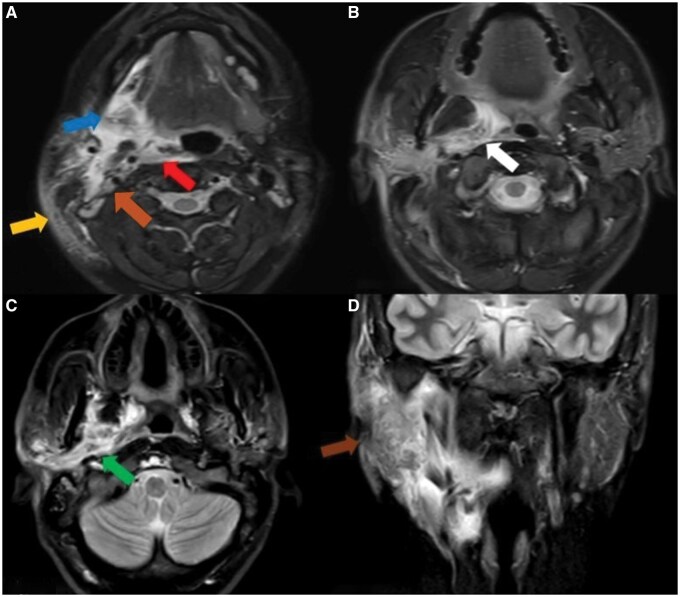
Fat-suppressed T2-weighted MR images demonstrating the multidirectional spread of the deep neck infection. (A) Axial fat-suppressed T2-weighted image showing subcutaneous extension (yellow arrow), involvement of the submandibular space (blue arrow), retro-pharyngeal spread (red arrow), and carotid space extension (orange arrow. (B) Axial fat-suppressed T2-weighted image depicting para-pharyngeal compartment involvement (white arrow). (C) Axial fat-suppressed T2-weighted image demonstrating spread into the masticator space (green arrow). (D) Coronal fat-suppressed T2-weighted image showing extension into the parotid compartment (brown arrow).

**Figure 3.**
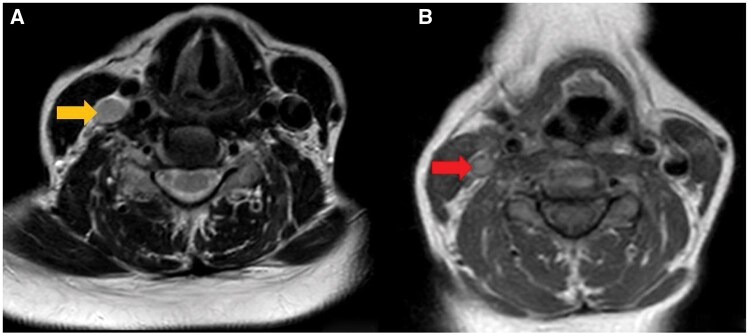
Axial non–fat-suppressed T1- and T2-weighted MR images illustrating findings consistent with Lemierre syndrome. (A) Axial non–fat-suppressed T2-weighted image demonstrating loss of the normal flow-related signal void within the right internal jugular vein (yellow arrow). (B) Axial non–fat-suppressed T1-weighted image similarly showing the absence of the expected venous signal void in the thrombosed right internal jugular vein (red arrow).

Imaging demonstrated loss of the normal flow void in the right middle temporal vein (Figure 1). There was edema in the subcutaneous tissues lateral to the right sternocleidomastoid muscle, together with extensive inflammatory changes involving the posterior cervical compartment, parotid compartment, masticator compartment, submandibular space, and parapharyngeal fat compartment. These inflammatory changes extended across multiple tissue planes, effacing normal fat boundaries and causing thickening and increased volume in the right parotid gland, right submandibular gland, and the muscles of mastication, including the medial and lateral pterygoid muscles. The inflammatory process encircled the carotid neurovascular bundle and resulted in medial displacement of the pharyngeal wall with secondary narrowing and deviation of the airway. Edema and soft tissue enhancement compatible with phlegmonous infiltration were also seen in close proximity to the retropharyngeal compartment (Figure 2). Loss of flow void in the right internal jugular vein was identified as well (Figure 3). These findings were consistent with middle temporal vein thrombophlebitis, cellulitis, myositis, multicompartment deep neck infection, and Lemierre syndrome developing in the early postoperative period following temporal lifting.

Broad-spectrum intravenous antibiotic therapy was initiated to target deep neck infection, and the regimen was optimized to provide high-dose coverage for both aerobic and anaerobic organisms, given the multicompartment extension and internal jugular vein involvement. Therapeutic-dose anticoagulation was started due to internal jugular vein thrombosis, with coagulation parameters monitored at regular intervals. Anticoagulation therapy was continued for a total duration of three months. Because edema and infiltration in the parapharyngeal region caused narrowing of the airway, the patient was placed under close respiratory observation. Oxygen saturation, respiratory effort, and pharyngeal patency were frequently reassessed, and preparations for emergent airway intervention were maintained.

With the combination of antimicrobial therapy, anticoagulation, and supportive management, the patient experienced complete clinical resolution of symptoms and findings.

## Discussion

This case illustrates a unique clinical scenario in which extensive infectious and vascular complications developed rapidly following an aesthetic surgical procedure performed in the temporal region. Considering the known anatomical patterns of deep neck infection spread, the fascial continuity between the temporal region and both the parapharyngeal and masticator compartments provides a potential conduit for downward extension. The deep neck spaces form a complex network of parapharyngeal, masticator, carotid, and retropharyngeal compartments interconnected by loose connective tissue and potential spaces, enabling rapid longitudinal and transverse propagation of infection.^6^^,^^7^ In the present case, the multicomponent infiltration observed on magnetic resonance imaging demonstrates that the inflammatory process originating in the temporal venous system had spread through these fascial planes to the lower cervical compartments. In particular, the infiltration of parapharyngeal fat resulting in airway narrowing indicates that such infections may progress quickly toward clinically significant and potentially life-threatening consequences.^6–8^

A central feature of this clinical presentation is the development of Lemierre syndrome with internal jugular vein involvement. The underlying pathophysiology involves microbial invasion of the venous endothelium, formation of platelet-rich thrombi, and subsequent septic embolization.^9^ Although it most commonly arises after oropharyngeal infections, cases secondary to traumatic or postoperative processes have also been described.^9^^,^^10^ In this patient, early loss of venous flow void within the internal jugular vein suggests postoperative regional infection–associated endothelial inflammation. The progression of thrombophlebitis from the temporal venous system toward the internal jugular vein is consistent with established anatomical venous drainage pathways, and the evolution into Lemierre syndrome reflects a sequential progression through well-defined pathophysiologic stages.

Diagnostic imaging, particularly magnetic resonance imaging, played a decisive role in establishing the diagnosis and guiding clinical management. Magnetic resonance imaging offers superior soft tissue contrast compared with computed tomography, enabling detailed visualization of fascial planes, muscular and glandular structures, deep neck compartments, and the venous system.^6–8^ In this case, the extensive inflammatory infiltration seen on postcontrast sequences provided clear delineation of the magnitude of the underlying process, including venous thrombophlebitis. Magnetic resonance imaging was also essential for differentiating between abscess and phlegmon and for accurately defining internal jugular vein involvement.^6–8^ This degree of diagnostic precision directly informed therapeutic strategy, supporting the early initiation of both antimicrobial therapy and anticoagulation.

Broad-spectrum antibiotic therapy remains the foundation of deep neck infection management, with strong emphasis on ensuring adequate anaerobic coverage.^6^^,^^9^ As in the present case, combined antibiotic regimens should be initiated promptly in the setting of multicompartment disease and subsequently adjusted according to clinical status and laboratory findings.^6^^,^^9^ In Lemierre syndrome, early administration of anticoagulation is considered important to prevent progression of thrombosis and to reduce the risk of septic emboli.^9^^,^^10^

The most striking aspect of this case is the demonstration, through magnetic resonance imaging, of an extensive inflammatory process arising after a superficially performed aesthetic procedure such as temporal lifting. The absence of prior reports describing either deep neck infection or internal jugular vein thrombophlebitis following temporal lifting highlights the unusual nature of this presentation. Nonetheless, radiologists should maintain a high index of suspicion for deep neck extension and venous involvement when evaluating postoperative inflammatory changes related to facial aesthetic surgery. Early imaging is particularly important in patients presenting with trismus, progressive pain, rapidly expanding edema, or difficulty swallowing, as these findings may signify deeper and clinically significant disease.

This case also has several limitations. First, additional imaging modalities such as ultrasonography or computed tomography were not used because they were not deemed necessary by the managing clinicians. As a result, the diagnostic assessment relied primarily on magnetic resonance imaging, precluding a multimodality evaluation. Second, follow-up imaging was not performed, and treatment response was assessed solely on clinical grounds. Thus, objective radiological data regarding the anatomical regression of infection or the evolution of the venous thrombosis were not available.

Despite these limitations, this case report demonstrates the feasibility and diagnostic value of a comprehensive magnetic resonance imaging assessment in identifying extensive infectious and vascular complications following temporal lifting. The imaging findings clearly reveal the role of magnetic resonance imaging in early diagnosis and effective clinical management. Based on the current literature review, no previously published report has described the concurrent occurrence of middle temporal vein thrombophlebitis, multicompartment deep neck infection, and Lemierre syndrome after temporal lifting. Accordingly, this case provides an important contribution to the literature by improving recognition of similar rare and potentially life-threatening conditions and by reinforcing the need for heightened clinical vigilance.

## Learning points

- Temporal lift may rarely lead to deep neck infection and Lemierre syndrome.
- Temporal infections may track along fascial planes into deep neck spaces.
- MRI is essential for detecting venous thrombosis and multicompartment spread.
- Post-lift trismus or swelling requires urgent evaluation for deep neck disease.
